# Non-conventional Yeast Species for Lowering Ethanol Content of Wines

**DOI:** 10.3389/fmicb.2016.00642

**Published:** 2016-05-04

**Authors:** Maurizio Ciani, Pilar Morales, Francesca Comitini, Jordi Tronchoni, Laura Canonico, José A. Curiel, Lucia Oro, Alda J. Rodrigues, Ramon Gonzalez

**Affiliations:** ^1^Dipartimento di Scienze della Vita e dell’Ambiente, Università Politecnica delle MarcheAncona, Italy; ^2^Instituto de Ciencias de la Vid y del Vino, Consejo Superior de Investigaciones Científicas-Universidad de La Rioja-Gobierno de La RiojaLogroño, Spain

**Keywords:** non-*Saccharomyces* yeasts, low alcohol wine, ethanol yield, yeast respiration, mixed starters

## Abstract

Rising sugar content in grape must, and the concomitant increase in alcohol levels in wine, are some of the main challenges affecting the winemaking industry nowadays. Among the several alternative solutions currently under study, the use of non-conventional yeasts during fermentation holds good promise for contributing to relieve this problem. Non-*Saccharomyces* wine yeast species comprise a high number or species, so encompassing a wider physiological diversity than *Saccharomyces cerevisiae*. Indeed, the current oenological interest of these microorganisms was initially triggered by their potential positive contribution to the sensorial complexity of quality wines, through the production of aroma and other sensory-active compounds. This diversity also involves ethanol yield on sugar, one of the most invariant metabolic traits of *S. cerevisiae*. This review gathers recent research on non-*Saccharomyces* yeasts, aiming to produce wines with lower alcohol content than those from pure *Saccharomyces* starters. Critical aspects discussed include the selection of suitable yeast strains (considering there is a noticeable intra-species diversity for ethanol yield, as shown for other fermentation traits), identification of key environmental parameters influencing ethanol yields (including the use of controlled oxygenation conditions), and managing mixed fermentations, by either the sequential or simultaneous inoculation of *S. cerevisiae* and non-*Saccharomyces* starter cultures. The feasibility, at the industrial level, of using non-*Saccharomyces* yeasts for reducing alcohol levels in wine will require an improved understanding of the metabolism of these alternative yeast species, as well as of the interactions between different yeast starters during the fermentation of grape must.

## Industrial And Social Interest in Reducing Alcohol Levels in Wine

The ethanol content in wine increased considerably over the past 20 years due to two main factors: the impact of climate change upon the global production of grapes, and the current quest for new wine styles, often requiring increased grape maturity (Jones et al., 2005; Grant, 2010; MacAvoy, 2010; Alston et al., 2011; Gonzalez et al., 2013). Late harvests are indeed required to meet present consumer’s preferences toward well-structured, full body wines, and optimal phenolic maturity of grapes. This practice results in a noticeable increase in the sugar content of the berries (Mira de Orduña, 2010) with consequent higher alcohol levels in wine. On the other hand, global climate change has deeply influenced the vine phenology and the grape composition, resulting in grapes with lower acidity, altered phenolic maturation and tannin content, and increasing sugar concentration (Jones et al., 2005). These changes further contribute to rising alcohol content in wines, in addition to modifying other wine sensory attributes as well as wine microbiology (Mira de Orduña, 2010). Alston et al. (2015) reported that the ethanol content in New World wines was higher than in European wines (13.65 vs. 13.01% v/v). The ethanol contents found in North American, Argentinean, Australian, and Chilean wines were 13.88, 13.79, 13.75, and 13.71% v/v, respectively. In Europe, Spain accounted the highest values (13.43% v/v). The high ethanol content in wine can lead to stuck and sluggish fermentations (Coulter et al., 2008) and to unbalanced wines that are unpleasant for consumers. Indeed, several studies reported that high ethanol concentration increase hotness and bitterness perceptions, while it decreases acidity sensations and masks the perception of some important aroma compounds such as higher alcohols, esters and monoterpenes (Escudero et al., 2007; Robinson et al., 2009; Fischer, 2010; Frost et al., 2015). This trend brings about some troubles for the wine industry, as well as social and public safety problems related to alcohol consumption (Grant, 2010; MacAvoy, 2010). In order to overcome these issues, the market focus is directed to wines with a moderate alcohol content. In addition, lowering ethanol content has an economic interest due to the high taxes imposed in some countries (Gil et al., 2013).

Pickering (2000) and Saliba et al. (2013) reported that wines with reduced ethanol content have been classified as dealcoholized or alcohol free (<0.5% v/v), low alcohol (0.5–1.2% v/v), reduced alcohol (1.2 to 5.5–6.5% v/v) and lower alcohol wine (5.5–10.5% v/v), even if these classifications, which are loosely based on labeling and legislative requirements, vary between different countries (Pickering, 2000). However, most winemakers are interested in developing practices aiming to reduce the alcohol concentration in wine by just 1–3% v/v, in order to compensate the impact of global warming and to obtain better-balanced wines (Meillon et al., 2010a,b; Gambuti et al., 2011). The winemaking industry is addressing this challenge by targeting almost all the different steps of the production cycle (Teissedre, 2013), starting from grapevine clonal selection, vineyard management, pre-fermentation and winemaking practices, microbiological approaches and post-fermentation and processing technologies (García-Martín et al., 2010; Gil et al., 2013; Poni, 2014; Varela et al., 2015).

In this regard, the viticultural practices to reduce ethanol content in wine act to manage grapes sugar content through different approaches such as reducing leaf area (defoliation or topping of shoots; Martinez de Toda et al., 2013; Poni, 2014), pre-harvest irrigation to cause a significant delay of ripening (Mendez-Costabel, 2007), application of growth regulators to postpone ripening (Symons et al., 2006) and manage harvest date (Bindon et al., 2013). At pre-fermentative stage the reduction of sugar concentration in must could be achieved by dilution of grape must with water (depending of country regulation) or using nanofiltration technologies (Harbertson et al., 2009; García-Martín et al., 2010). Another pre-fermentative strategy to remove sugar from must could be the addition of glucose oxidase enzyme (Pickering, 2000). The ethanol reduction in wine could be also achieved at post-fermentation stage. In this regard, it is possible to mention the blending of low-high alcohol wines or physical removal of alcohol from wine with membrane-based system, vacuum distillation and supercritical CO_2_ extraction (Gambuti et al., 2011; Kontoudakis et al., 2011; Schmidtke et al., 2012).

## *S. cerevisiae* is not the Best Yeast Species for Reducing Alcohol Levels in Wine

Development and application of yeast strains showing below normal alcohol production has been a recurrent objective for wine biotechnology for more than 20 years, starting even before increasing ethanol content in wines was widely perceived as a problem. Low alcohol production by yeasts might be related with two distinct metabolic features, alcohol tolerance, or ethanol yield on sugar. Traditional scientific literature on wine yeast often use the term fermentative power, to refer to the amount of alcohol produced by different yeast strains from natural or synthetic grape must (Lopes et al., 2006). Due to the assay conditions, this parameter is mainly related to alcohol tolerance, and tells little about the usefulness of yeast strains for alcohol level reduction. Indeed, oenological use of yeast strains having low fermentative power would result in either stuck fermentation or the starter being quickly replaced by native yeasts.

To attain a relevant alcohol level reduction in wine (fermented to dryness), the appropriate yeast metabolic trait to take into account is alcohol yield on sugar. Ethanol yield on sugar is formally expressed as grams of ethanol produced per gram of glucose or fructose consumed (g/g). The rule of thumb says consumption of 17 g/L of sugar will result in an increase of 1% v/v in alcohol content. Not surprisingly, being *Saccharomyces cerevisiae* the main yeast species responsible of alcoholic fermentation during winemaking, it has almost invariably been the species of choice for all research efforts aiming to reduce ethanol yields. However, evolution has shaped this species to quickly and efficiently produce ethanol from sugars under most environmental conditions, following the make-accumulate-consume life strategy (Piskur et al., 2006). Although, some natural variability can be found among wild isolates of this species, the distribution of ethanol yield values is rather narrow (around the values mentioned above).

**FIGURE 1:**
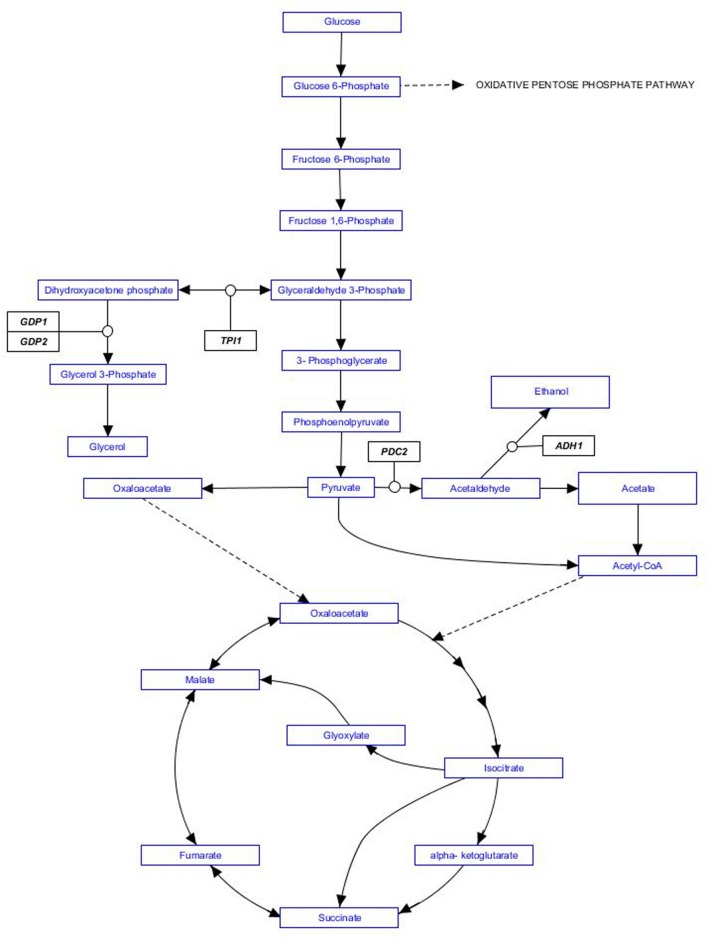
**Metabolic pathways involved in ethanol production by *Saccharomyces cerevisiae*.** Genes targeted by genetic engineering strategies mentioned in the text are indicated in black boxes.

Researchers have designed several alternative genetic engineering approaches in order to partially redirect *S. cerevisiae* normal carbon flux, starting with the overexpression of *GPD1* or *GPD2*, coding for isozymes of glycerol 3-phosphate dehydrogenase. The choice of *GPD* genes was additionally driven by glycerol contribution to sweetness, smoothness and wine body. Other strategies aiming to reducing alcohol yields also involve genetic manipulation of the central carbon and energy metabolism of *S. cerevisiae*. Target genes include for example *PDC2*, coding for pyruvate decarboxylase; *ADH1*, coding for alcohol dehydrogenase; or *TPI1*, coding for triose phosphate isomerase. An excellent review by Kutyna et al. (2010) gathers additional genetic engineering strategies in order to reduce alcohol yield during wine fermentation. However, a recent experimental evaluation of genetic modifications to develop low ethanol yield wine yeast strains concluded that overexpression of *GPD1* was the most efficient strategy to lower alcohol yield (Varela et al., 2012). Also in agreement with early studies in this field (Remize et al., 1999; Cambon et al., 2006) they found overexpression of *GPD1* resulted in the overproduction of some metabolites negatively affecting wine quality. In order to avoid these drawbacks additional genetic modifications were required (Cambon et al., 2006; Ehsani et al., 2009). Metabolic pathways mentioned in this paragraph are summarized in **Figure 1**.

**FIGURE 2:**
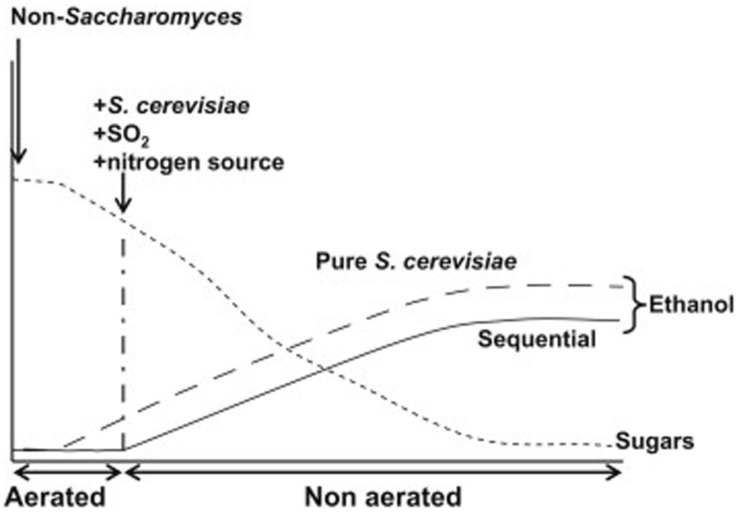
**Idealized representation of the expected evolution of ethanol production during grape must fermentation in a sequential inoculation with a Crabtree-negative non-*Saccharomyces* yeast strain, followed by *S. cerevisiae* at the moment indicated (continuous line).** Aeration would be restricted to the first stages of alcoholic fermentation, as indicated. The expected evolution of ethanol production for a pure *S. cerevisiae* starter in the same conditions is indicated by a dashed line. For simplicity, sugar consumption has been assumed to follow a similar pattern in both situations. Reproduced from Gonzalez et al. (2013) with permission of the copyright owner.

Limitations of the genetic engineering approach are twofold. First, commercial use of genetically engineered wine yeast strains does not seem to be feasible in the short term (Gonzalez et al., 2013). In order to circumvent this problem, some researchers are now using adaptive laboratory evolution (Cadière et al., 2011; Kutyna et al., 2012). Second, the increase in concentration required to reach a relevant impact on wine final alcohol content (2–3% v/v), would certainly compromise wine quality for most alternative metabolites. This holds true even for glycerol, one of the preferred targets for researchers in this field. Reduction of 2% v/v ethanol by diverting carbon flux toward glycerol production would result in more than 30 g/L extra glycerol (about five times the usual values). Almost any other chemical compound would also become unacceptable in wine at such elevated concentrations. Carbon dioxide is perhaps the only metabolite that would cause no trouble when overproduced by yeast during wine fermentation, in part because it is readily released to the atmosphere. The two main metabolic pathways for CO_2_ production are respiration and fermentation. Concerning alcohol reduction, the advantage of respiration is that no ethanol is produced, since all six carbon atoms from each molecule of sugar end up as CO_2_. Some researchers have suggested partial respiration of sugars from grape must as a way to decrease ethanol yield during winemaking (Gonzalez et al., 2013 and references therein). A possible way to reach this goal is shown in **Figure 2**.

There are, however, two restrictions to make yeast cells respire sugars under standard winemaking conditions, oxygen requirement and the Crabtree effect. Respiratory metabolism has a huge oxygen demand, but it is known to participate in many other chemical reactions that can be detrimental to wine quality. Proper management of dissolved oxygen during wine fermentation will be required in order to meet respiration requirements while preserving other wine compounds from excessive oxidation (see below). On the other side, *S. cerevisiae* is the archetype Crabtree-positive yeast species. This metabolic feature strongly favors fermentative over respiratory metabolism, despite oxygen availability (Pronk et al., 1996), and have played a key role in the adaptation of this species to sugar rich environments (Piskur et al., 2006). In *S. cerevisiae* “aerobic fermentation” involves usually above 98% of the sugars consumed in the presence of oxygen (de Deken, 1966). Only under conditions of very low sugar availability (which is not obviously the case for grape must), is respiration the main energetic metabolic pathway in this species (Pronk et al., 1996). The possibility of reducing ethanol yields by promoting respiration of sugars by *S. cerevisiae* or other yeast species was initially suggested by Smith (1995), and the idea has been independently recovered and developed to different levels in recent years (Erten and Campbell, 2001; Contreras et al., 2015b; Morales et al., 2015; see below).

## Sugar Metabolism of NS Yeasts

Common ethanol yields on sugar after complete grape juice fermentation are 90–95% of theoretical, with the remaining 5–10% being explained by biomass biosynthesis, ethanol stripping, and alternative metabolic pathways (Konig et al., 2009). This mainly reflects anaerobic carbon flux distribution in *S. cerevisiae*. However, NS wine yeasts usually differ from *S. cerevisiae* in metabolic flux distribution during fermentation and, consequently, in ethanol production, biomass synthesis, and by-product formation (Ciani et al., 2000; Magyar and Toth, 2011; Milanovic et al., 2012; Tofalo et al., 2012). Under anaerobic conditions, the diversion of alcoholic fermentation and the abundant formation of secondary compounds may in part explain the low ethanol yield of some of these NS yeast species/strains. Indeed, some of these species are strongly characterized by species-specific patterns of fermentation by-products, which allows the differentiation of the majority of these yeast strains according to the species (Domizio et al., 2011).

The production of ethanol and the other main fermentation compounds are metabolically linked. In *S. cerevisiae* glycerol production is highly correlated with the production of acetic acid (Ciani and Rosini, 1995). Indeed, as mentioned above, genetic engineering of *S. cerevisiae* for glycerol overproduction often results a large production of acetic acid (Remize et al., 1999, 2000; Eglinton et al., 2002). The evaluation of the relation between fermentation by products and ethanol production among several NS wine yeasts revealed both direct and inverse correlations between acetic acid and ethanol production, for *Saccharomycodes ludwigii* and *Kloeckera apiculata*, respectively (Ciani and Maccarelli, 1998). In contrast, *Torulaspora delbrueckii*, *Candida stellata*, and *Hanseniaspora uvarum* did not show any correlation between the two fermentation products. Ethanol is positively correlated with glycerol and ethyl acetate in *C. stellata* and *K. apiculata* respectively, while an inverse correlation between ethanol and succinic acid production was shown for *T. delbrueckii*.

The most striking metabolic trait of *S. cerevisiae* is perhaps the Crabtree effect. This feature makes *S. cerevisiae* preferentially consume sugars by fermentation in almost any growth condition, apart from carbon limited chemostat operated at low dilution rates. This trait has been often related to glucose triggered transcriptional repression of genes involved in respiratory functions (Barnett and Entian, 2005). However, current understanding of the Crabtree effect points to overflow metabolism at the level of the pyruvate node, as the main mechanism contributing to the observed distribution of carbon flux toward ethanol production (Holzer, 1961; Pronk et al., 1996). In addition, Aceituno et al. (2012) found cytoplasm-to-mitochondria NADH transport to be a limiting factor to get a fully aerobic metabolism in the presence of oxygen. Independent of the mechanism, the critical factor determining the respiro-fermentative balance in *S. cerevisiae* seems to be the rate of sugar consumption. Indeed, mutations slowing down the glycolytic rate result in a noticeable relief of the Crabtree effect (Otterstedt et al., 2004; Jansen et al., 2005).

Several classifications of yeast species, according to the way they regulate respiro-fermentative metabolism have been proposed (Gancedo and Serrano, 1989; Alexander and Jeffries, 1990). In general, they are categorized as either Crabtree-positive or Crabtree-negative, or as obligate respiratory. Assessment of the Crabtree status is generally based on studies under carbon limited chemostat conditions (Pronk et al., 1996). So, despite most yeast species found in the oenological environment have shown fermentative capacity (Kurtzman et al., 2011), most of them have never been evaluated for Crabtree status. Furthermore, according to recent studies (Quirós et al., 2014; Contreras et al., 2015b) the classification based on standard Crabtree assays has little prediction power on the behavior of yeasts under growth conditions more closely mimicking those found in wine fermentation. In addition, important differences can be found among yeast strains belonging to the same species.

Analysis of the respiro-fermentative behavior of yeast strains under controlled aeration conditions in high sugar containing media has usually confirmed *S. cerevisiae* as one of the most fermentation-prone yeast species. However, strains from some other species have shown even higher ethanol yield or RQ values than control *S. cerevisiae* yeast strains under such assay conditions (Quirós et al., 2014; Contreras et al., 2015b). Interestingly, respiratory behavior of yeast strains seems to be strongly affected by other environmental factors, not only sugar abundance or oxygen availability (Rodrigues et al., 2016). The extent to which these environmental factors affect yeast respiro-fermentative metabolism, and secondary by-products like glycerol or acetic acid, is species or strain-specific. Further research is required in order to understand the metabolic diversity of NS yeast species and the relevance of this diversity for oenological applications, including reducing ethanol content of wines.

## Selection of Non-*Saccharomyces* Wine Yeast to Reduce the Ethanol Content

During wine production, the non-*Saccharomyces* (NS) yeasts contribute to the fermentation process, either directly or through their effect on both growth kinetics and metabolic activity of *S. cerevisiae* (Ciani and Comitini, 2015). These NS yeasts are capable of anaerobic or aerobic growth and may persist during the fermentation, competing with *Saccharomyces* for nutrients, producing secondary compounds or modifying the *S. cerevisiae* metabolism (Milanovic et al., 2012; Sadoudi et al., 2012; Barbosa et al., 2015).

NS wine yeasts have been shown to modulate wine fermentation and to enhance sensorial complexity and aroma profile of wines (Fleet, 2008; Ciani et al., 2010). In addition, some of these NS species/strains are able to combat spoilage yeasts (Comitini et al., 2011; Oro et al., 2014; Alonso et al., 2015). Thus, over the last years, the role of NS yeasts in winemaking, previously neglected or demonized, has been re-evaluated, and their use has been proposed in controlled mixed fermentation with the aim to improve wine complexity, aroma profile and control of spoilage microorganisms (Rojas et al., 2001; Swiegers et al., 2005; Domizio et al., 2007; Renouf et al., 2007; Anfang et al., 2009; Comitini et al., 2011; Jolly et al., 2014). In this context, the metabolic traits of NS wine yeasts could also be profitably used to reduce the alcohol content in wine. This application would benefit from a better understanding of the metabolic pathways diverting carbon flux from ethanol production in NS yeasts, as well as the biological variability of these yeast species in terms of ethanol yield. One of these alternative pathways would be sugar respiration under suitable fermentation conditions, especially for Crabtree-negative yeast species, as discussed in other sections of this review. In summary, our current knowledge suggests several promising approaches for the use of NS wine yeast to limit ethanol production. However, taking into account the current interest on NS wine yeasts is mostly related to their impact on wine sensory quality (Lambrechts and Pretorius, 2000; Romano et al., 2003; Ciani et al., 2010; Belda et al., 2015, 2016; Wang et al., 2015; Hu et al., 2016; Masneuf-Pomarede et al., 2016; Medina et al., 2016), a positive contribution to wine aromatic complexity would certainly be a plus in yeast strain selection for this purpose.

### Screening Based on Low Ethanol Yield under Anaerobic Fermentation Conditions

Over the recent years, there was a rising interest to investigate on the wine yeast variability in ethanol yield as a potential tool for the reduction of alcohol content in wine. Variability between different yeasts genera and species could be exploited at industrial level to produce wines better fitting consumer preferences. Reduction in ethanol yield is strictly dependent on the microbial strategies that divert sugar-carbon away from ethanol production.

As mentioned above, *S. cerevisiae*, shows high fermentation performance with high ethanol yield and fermentation efficiency, exhibiting a low intraspecies variability for these characters. In contrast, NS wine yeasts show, as a trend, lower ethanol production and lower ethanol resistance. Overall these features are considered to be a major factor of the dominance of *S. cerevisiae* over NS species during wine fermentation. Generally, the species belonging to *Hanseniaspora, Candida, Pichia, Kluyveromyces, Metschnikowia*, *Torulaspora*, and *Issatchenkia* genera, widely or occasionally found in grape juice, are not tolerant to ethanol concentrations above 5–7% v/v. Their decline and death as the fermentation progresses can be mostly explained by their low alcohol tolerance, even though recent studies indicate that the interactions with *S. cerevisiae* might be more complex (Arneborg et al., 2005; Branco et al., 2014; Ciani and Comitini, 2015). On the other hand, NS wine yeasts exhibit a broad spectrum of fermentation by-products, low fermentation purity (volatile acidity g/L ÷ ethanol % v/v) and, often, low ethanol yield (Muller-Thurgau, 1896; Ribéreau-Gayon and Peynaud, 1960; Romano et al., 1992). A systematic investigation on fermentation by-products formed by a wide collection of NS wine yeasts, belonging to five different species, was carried out by Ciani and Maccarelli (1998). In that work, “apiculate” yeast species showed a high production of acetaldehyde, ethyl acetate and acetoin; *C. stellata* exhibited high production of glycerol and succinic acid, while *T. delbrueckii* was shown to be a lower producer of secondary products of fermentation. In the various NS species tested, the ethanol production is differently related with the fermentation by-products. NS wine yeasts are generally low-ethanol producing yeasts. However, this feature does not necessarily mean that they exhibit also low ethanol yield.

In this context, only recent studies addressed the interspecies and/or intraspecies variability in ethanol yield among NS wine yeasts (Magyar and Toth, 2011; Contreras et al., 2014, 2015b; Gobbi et al., 2014). In a comparative evaluation of some oenological properties in several wine strains, Magyar and Toth (2011) found a very low ethanol yield for four *Candida zemplinina* strains. Gobbi et al. (2014), investigating on several NS wine yeast species, showed that strains belonging to the species of *H. uvarum*, *Zygosaccharomyces sapae*, *Zygosaccharomyces bailii*, and *Zygosaccharomyces bisporus* exhibited significant low ethanol yield and fermentation efficiency in comparison with *S. cerevisiae* under anaerobic conditions and using different grape juices. For *H. uvarum*, these data confirm the low ethanol yield previously described (Ciani et al., 2006), in contrast to species belonging to the *Zygosaccharomyces* genus. Moreover, they found that ethanol yield, like other fermentation features, is a species-related trait. However, as indicated previously for other fermentation parameters (Ciani and Maccarelli, 1998; Comitini et al., 2011; Domizio et al., 2011), a pronounced intraspecies variability was also evident. In another recent work a screening on 50 different NS strains belonging to 24 different genera for their ethanol yield was carried out (Contreras et al., 2014). This led to the identification of four NS yeast strains (two strains of *Metschnikowia pulcherrima* and one strain each of *Schizosaccharomyces malidevorans* and *C. stellata*) that showed low ethanol yield. In a different study, under semi-aerobic condition, nine out of 48 NS strains showed ethanol yields lower than the *S. cerevisiae* control strain. Three of them (*T. delbrueckii* AWRI1152, *Pichia kudriavzevii* AWRI1220, and *Z. bailii* AWRI1578) gave promising results in the subsequent aerobic sequential trials, with *S. cerevisiae* AWRI1631 (Contreras et al., 2015b; see below for further discussion on this work).

Some *Saccharomyces* species, other than *S. cerevisiae*, have also shown potential for ethanol reduction. This is the case for *Saccharomyces uvarum*, a cryophilic species that has been described as a low ethanol and high glycerol producer (Giudici et al., 1995). Fermentation kinetics in must at 13°C is better for *S. uvarum* than for *S. cerevisiae*, but some strains get stuck at 8% v/v alcohol when run at 24°C (Kishimoto et al., 1994; Masneuf-Pomarede et al., 2010). In a sequential inoculation of *S. uvarum* (AWRI 2846) and *S. cerevisiae*, Contreras et al. (2015a) found an ethanol reduction of 0.8% v/v, and an increase of glycerol of 6.4 g/L. The decrease in ethanol production was not fully explained by the increase in glycerol, in terms of carbon mass balance.

### Respiration Based Screening

As mentioned above, development of respiration based methods to reduce alcohol content in wine requires the use of NS yeast strains showing no or weak Crabtree effect. However, this metabolic feature, which is indeed rather common across the yeast phylogeny (de Deken, 1966), is not sufficient to warrant the utility of a given yeast species/strain for such purpose. Suitable yeast strains must be able to develop in grape must, a relatively harsh growth medium due to osmotic stress, low pH, and the presence of natural or added inhibitors of microbial growth. In addition sugar consumption kinetics should be relatively fast, in order to be compatible with industrial procedures; as well as being able to dominate fermentation processes, in competition with the microbiota naturally present in grape must. Finally, they must not generate secondary metabolic products that would result in wine spoilage, in either aerobic or anaerobic conditions.

Initial trials to follow the sugar respiration strategy analyzed the behavior of three to four yeast strains in synthetic or natural grape juice under aerobic or microaerobic conditions (Smith, 1995; Barwald and Fischer, 1996; Erten and Campbell, 2001). More recent studies use a higher number of yeast strains (around 60) and milder aeration regimes, than previous studies (Quirós et al., 2014; Contreras et al., 2015b). Quirós et al. (2014) chose respiratory quotient (RQ) as an indicator of the respiration capabilities of each yeast strain. RQ can be calculated as the ratio of CO_2_ produced to O_2_ consumed. When hexoses are used as substrate RQ can range from 1 (full respiration) to ∞ (full fermentation). The relationship between RQ and the percentage of sugar consumed by respiration (%SR) can be expressed as follows: %SR = 100/(3RQ-2). They calculated RQ values, in synthetic medium containing 200 g/L sugar, pH 3.5, and high biomass content (OD600 = 20), under strongly aerated conditions, and identified strains from several yeast species with RQ values close to 1 under these specific growth conditions. The advantage of RQ over direct calculation of ethanol yields is it is not affected by ethanol stripping. One alternative which is especially valid for mild aeration regimes is comparing ethanol yields with *S. cerevisiae*, in order to identify low yield candidates, and setting control experiments with pure nitrogen gas at the same flow rate, in order to compare ethanol yields between anaerobic and aerobic (or micro-aerobic) conditions (Contreras et al., 2015b; Morales et al., 2015). We must, however, stress that Contreras et al. (2015b) considered their aeration conditions to be not strong enough to trigger respiratory metabolism.

However, low respiratory quotient or low ethanol yields are not enough to ensure the usefulness of yeast strains for the purposes discussed in this review. Indeed, strains showing a strong preference for respiratory metabolism would be completely useless if the amount of sugar they metabolized were negligible (in a reasonable fermentation time). Hence, authors took into account sugar consumption after 3 or 4 days on synthetic grape must in order to identify interesting strains (Quirós et al., 2014; Contreras et al., 2015b).

The other main aspect to be taken into consideration for a proper yeast strain selection in this context is volatile acidity. There are already several reports showing an important increase in acetic acid yield for *S. cerevisiae* under aerated conditions, as compared to anaerobic growth (Giovanelli et al., 1996; Papini et al., 2012; Quirós et al., 2014; Contreras et al., 2015b; Rodrigues et al., 2016). Strains from other yeast species have also been found to produce high amounts of acetic acid under oxygenation (Quirós et al., 2014; Contreras et al., 2015b); and some of them also under standard fermentation conditions (Ciani and Picciotti, 1995; Viana et al., 2008).

## Managing Mixed Fermentations

Apart from reducing ethanol yields, the main driver for the current development of NS commercial starters is related to the increasing consumer demand for wines showing improved sensorial properties and distinctive flavor (Pretorius and Hoj, 2005; Belda et al., 2015, 2016; Masneuf-Pomarede et al., 2016; Medina et al., 2016), in contrast to the limited complexity attributed to wines fermented with *S. cerevisiae* starter strains (Heard, 1999; Rojas et al., 2003; Romano et al., 2003; Ciani et al., 2006, 2010; Jolly et al., 2006). However, NS wine yeasts often show low fermentation power. For this reason *S. cerevisiae* starters have to be used to ensure consumption of all sugars from grape must, and to bring the fermentation process to completion. In addition, the interactions between *Saccharomyces* and NS yeasts can be exploited to modulate the content of ethanol in wine (Ciani and Comitini, 2015; Wang et al., 2015). Temperature, sulphite content, sugar concentration, nitrogen composition, oxygen and pH, which influence glycerol and ethanol biosynthesis, must also be modulated and controlled.

Mixed starters can be used by either simultaneous or sequential inoculation. This later modality allows to take advantage of the metabolism of the first inoculated NS yeast without the influence of the *Saccharomyces* starter culture. In this way, the reduction in ethanol content will depend on the metabolic characteristics of the NS strain used, and on the actual opportunity it will have to stamp its metabolic footprint before *S. cerevisiae* takes over. Some important control parameters should be taken in account for this purpose: the inoculation concentration and the timing between the first and second inoculation, nutrient consumption and sulphite content. High inoculation level of NS yeast improves the competitiveness toward *S. cerevisiae* and other wild yeasts; while the interval between the first and the second inoculation affects the duration of this metabolic activity, which will quickly decline upon inoculation of *S. cerevisiae*. However, attention must also be paid to the consumption of nitrogen sources and vitamins from grape must by NS yeasts during the first stage of sequential inoculation fermentation (Kemsawasd et al., 2015). This consumption often requires to be compensated by suitable yeast nutrients in order to prevent stuck fermentations after inoculation of *S. cerevisiae* (Medina et al., 2012; Lage et al., 2014). Special attention is required in oxygenated fermentations, since a strong nutrient depletion is expected due to high biomass production by NS yeasts under these growth conditions. Concerning sulphite concentration, it must be adjusted during the first stage to the actual tolerance of the NS yeast strain used, since it will usually fall below the standard values for *S. cerevisiae* strains. Eventually, they might be raised to ordinary winemaking concentration after the second inoculation. Interestingly, controlled fermentation by sequential inoculation has been proposed as a way to reduce sulphite contents in the final wine.

The sequential inoculation strategy, using NS/*S. cerevisiae* has been employed in several studies. Many of them use *Starmerella bombicola* (formerly *C. stellata*) as the NS counterpart to *S. cerevisiae*. In these investigations a high production of glycerol and succinic acid and interactions involving some by-products (acetaldehyde, acetoin) with a consequent reduction of final ethanol amount were found (Ciani and Ferraro, 1996, 1998; Ferraro et al., 2000). The reduction in ethanol content in these assays varied from 0.64% v/v at pilot scale in natural grape juice to 1.60% v/v at laboratory scale using synthetic grape juice. Sequential fermentation trials using *Lachancea thermotolerans* (formerly *Kluyveromyces thermotolerans*) were carried out under industry condition using a high inoculation level (10^7^ cell/ml) with a delay of the second inoculum (*S. cerevisiae* strain) of 2 days resulting in an ethanol reduction of 0.7% v/v (Gobbi et al., 2013). A sequential inoculation of *M. pulcherrima* AWRI1149 followed by a *S. cerevisiae* wine strain gave rise to a wine with an ethanol concentration lower than that achieved with *S. cerevisiae* (0.9 and 1.6% v/v in Chardonnay and Shiraz wines, respectively; Contreras et al., 2014). Di Maio et al. (2012) showed that *C. zemplinina* may be used in mixed fermentation with *S. cerevisiae* to reduce the ethanol content in wine (0.32% v/v) and to increase the glycerol content More recently, the use of sequential fermentation with immobilized non-*Saccharomyces* wine yeast, was proposed to reduce the ethanol content in wine using *S. bombicola*, *M. pulcherrima*, *H. uvarum*, and *Hanseniaspora osmphila* selected strains, in Verdicchio grape juice. Sequential fermentation of 72-h showed an ethanol reduction of 1.64% (v/v) for *S. bombicola*, 1.46% (v/v) for *M*. *Pulcherrima*, 1.21% (v/v) for *H. uvarum*, and 1.00% (v/v) for *H. osmophila.* The wines obtained did not exhibit any negative fermentation products, but rather an increase of some desirable compounds (Canonico et al., 2016). In **Table 1** are summarized the anaerobic sequential fermentations of some NS yeasts as compared to control *S. cerevisiae* proposed to reduce the ethanol content in wine.

**Table 1:** Reduction of ethanol content in anaerobic sequential fermentations of some NS yeasts as compared to control *Saccharomyces cerevisiae*.

Sequential fermentation	Grape juice	Ethanol reduction % (v/v)	Reference
*S. bombicola/S. cerevisiae*	White	0.64	Ferraro et al., 2000
*S. bombicola/S. cerevisiae*	Synthetic	1.60	Ciani and Ferraro, 1998
*S. bombicola/S. cerevisiae*	White	1.64	Canonico et al., 2016
*H. uvarum/S. cerevisiae*	White	1.21	Canonico et al., 2016
*H. osmophila/S. cerevisiae*	White	1.00	Canonico et al., 2016
*M. pulcherrima/S. cerevisiae*	White	0.90	Contreras et al., 2014
*M. pulcherrima/S. cerevisiae*	Red	1.60	Contreras et al., 2014
*M. pulcherrima/S. cerevisiae*	Red	0.90	Contreras et al., 2015a
*M. pulcherrima/S. cerevisiae*	White	1.46	Canonico et al., 2016
*L. thermotolerans/S. cerevisiae*	Red	0.70	Gobbi et al., 2013
*C. zemplinina/S. cerevisiae*	Red	0.32	Di Maio et al., 2012

As a general trend, using NS/*S. cerevisiae* pairs in mixed fermentation did not result in the overproduction of undesirable by-products, in contrast to some *S. cerevisiae* genetically engineered strains, which can dramatically accumulate acetic acid or other metabolites with a negative impact on wine sensorial quality (Michnick et al., 1997; Remize et al., 1999). Indeed, apart from the reduction of ethanol content in wine, positive interactions in fermentation by-products have been shown during sequential fermentation. In NS/*S. cerevisiae* mixed cultures, the interactions due to the wide inter-generic metabolic diversity should be higher. These interactions were investigated in *S. cerevisiae* and *S. bombicola* (Sipiczki et al., 2005) mixed fermentation. In this co-culture complementary consumption of glucose and fructose was observed (Ciani and Ferraro, 1998). Using sequential, continuous fermentation and immobilized yeast cells, preliminary evidence has highlighted the exchange of acetaldehyde between these two yeast species. The excess of acetaldehyde production by *S. bombicola*, due to the low activity of alcohol dehydrogenase (Ciani et al., 2000), was quickly metabolized by *S. cerevisiae*, which is a more active alcoholic fermentation species (Ciani and Ferraro, 1998). In this context, an acetaldehyde flux between *S. cerevisiae* and *Saccharomyces bayanus* has also been reported (Cheraiti et al., 2005). These interactions in acetaldehyde reduction, were also detected in mixed fermentations using *S. cerevisiae*, *T. delbrueckii* (Ciani et al., 2006; Bely et al., 2008; Belda et al., 2015) and *L. thermotolerans* (Ciani et al., 2006). Another compound involved in interactions between two yeast species in mixed fermentation is acetoin; this is largely accumulated by *S. bombicola* in pure culture, and completely metabolized by *S. cerevisiae* in mixed fermentation (Ciani and Ferraro, 1998). More recently, the *influence Hanseniaspora guilliermondii* on genomic expression of *S. cerevisiae* in mixed culture wine fermentation was investigated (Barbosa et al., 2015)

On the other hand, oxygenated fermentation, as proposed above to stimulate yeast respiration, introduce a new challenge for managing mixed fermentations. Oxygen supply has a positive impact in several microbial and chemical processes during winemaking. It activates *S. cerevisiae* metabolism, in part because it is required for the biosynthesis of plasma membrane sterols, so aeration practices are often used in order to ensure good initial fermentation kinetics or to help recover sluggish fermentation (Alexandre and Charpentier, 1998; Valero et al., 2001; Fornairon-Bonnefond et al., 2002). Oxygen is also used in hyper-oxygenation treatments, in order to get rid of compounds highly sensitive to oxidation that would contribute to browning of white wines if oxidized in later stages of the winemaking process. In turn, macro- and micro-oxygenation of wines are used, alone or in combination with other oenological practices, in order to improve and stabilize wine color during the aging of red wines, or to avoid the “reduced” character sometimes associated to aging on yeast lees (Fornairon-Bonnefond et al., 2003).

Nevertheless, oxygen supply amounts required to ensure efficient yeast respiration are far beyond requirements for even the most demanding oxygenation practices, among those described above. There is a risk that the strong oxygenation levels required for yeast respiration would promote, as a side effect, the oxidation of key components for the sensory quality of wines, namely phenolics and aroma compounds. However, oxygen affinity of fermenting yeast cells has been determined to be about 1000 times higher than wine polyphenols (Salmon, 2006). Accordingly, the target to avoid oxidative damage to wine phenolics would be coupling air supply to oxygen consumption by yeast cells. Being able to keep dissolved oxygen values around 0% would be a good indicative of success for this objective. This goal was shown to be feasible by using controlled aeration conditions and an appropriate *M. pulcherrima* strain (Morales et al., 2015).

An additional major issue of strong aeration of wine during the fermentation step is acetic acid production. Several authors have described a boost in acetic acid production by *S. cerevisiae* when fermenting under aerobic or micro-aerobic conditions (Giovanelli et al., 1996; Papini et al., 2012; Quirós et al., 2014; Contreras et al., 2015b; Rodrigues et al., 2016). Other yeast species have also been shown to negatively impact volatile acidity under aerated growth conditions in synthetic grape must (Quirós et al., 2014). Rodrigues et al. (2016) analyzed volatile acidity across several growth conditions for four different yeast strains. They found a clear correlation between oxygen supply and acetic acid production. The good news is that some yeast species produce very little volatile acidity even under oxygenated conditions (Quirós et al., 2014; Rodrigues et al., 2016). It is possible to manage oxygen supply in fermentation trials driven by simultaneous inoculation of *S. cerevisiae* and a NS strain (Morales et al., 2015). However, the strict control of the process required under such growth conditions suggests that a better control of volatile acidity would be achieved by inoculating *S. cerevisiae* only after oxygen supply has been arrested (i.e., by sequential inoculation). In addition, proper control of yeast metabolism in aerated fermentations would benefit from the development of dedicated devices, able to monitor oxygen consumption and to adapt air supply to yeast requirements, so avoiding both excess oxidation and excess acetic acid production.

## Conclusion

Research on yeast based strategies in order to reduce ethanol content of wines started about 20 years ago (Michnick et al., 1997). The growing evidence about global climate warming and its impact on sugar content of grapes at harvest has contributed to an ever increasing interest on this topic. The biotechnological strategies initially explored were based on genetic engineering of *S. cerevisiae*, a rational choice considering the preponderant role of this species in both spontaneous and inoculated fermentation. However, the use of these recombinant strategies soon faced hurdles coming from both yeast metabolism and the regulatory framework for genetically modified organisms. The fact that further genetic modification was required in order to overcome initial problems did not help much.

In this context, the intense research activity around NS wine yeasts, our increasing awareness about the metabolic diversity of yeasts, and the arrival to the market of NS starters, opened new opportunities to exploit yeast metabolism with the aim of reducing ethanol content of wines. Current knowledge indicate that, similar to other metabolic traits, ethanol yield on sugar is not only species-specific, but often strain-specific. Some NS yeast species can show ethanol yields similar or higher than *S. cerevisiae*, but many of them show reduced ethanol yields. It has also been shown that oxygenation during wine fermentation can help further reduce ethanol yields by these, often Crabtree negative, NS yeast species.

Since most NS wine yeasts are sensitive to ethanol concentrations above 6–8%, in order to keep the microbiological control of the fermentation process, and to avoid stuck or sluggish fermentation (and wine spoilage), the use of *S. cerevisiae* starters, in either sequential or simultaneous inoculation, will still be required. The introduction of mixed starter inoculation to routine winemaking practices also demands for a better control of the fermentation parameters, adapted to each specific combination of yeast starters. Some parameters to take into account are sulphite concentration, temperature, pH adjustments, inoculation levels (and timing, for sequential inoculation), yeast nutrition, and eventually oxygenation levels and timing, among other parameters.

In order to perform knowledge based decisions in this field, further research will be required. Some of the topics that need to be addressed are common to other oenological applications of NS wine yeasts, while other are more specific for alcohol level reduction. Environmental factors influencing ethanol yield by different wine yeast species warrant a special attention, both for respiration-based and anaerobic fermentation strategies. As a general rule, research projects on NS wine yeasts should always pay attention to the production of unwanted metabolites, including acetic acid, which has been identified as a serious drawback, especially for respiration-based strategies or certain yeast strains. Reliable assessment of the impact of new yeast strains/species, and new oenological practices on quality related features of wines would require pilot scale experiments, use of natural grape must, and rigorous sensory analysis. One complex but very relevant aspect that is indeed already attracting attention by wine biotechnologists is physiological and ecological interactions between cells from the starter cultures, among them and with the natural microbiota. The few articles already published on this topic are just opening the window to a world of interactions, including competitions, metabolite exchanges, and production of narrow and wide spectrum antimicrobials. All these phenomena have a potential to impact alcohol level and overall quality of wines. Yeast inter and intraspecific diversity must always be taken into account both in the design of experiments and to draw general conclusions. Finally, the interaction of starter cultures with natural microbiota is a very relevant but complex topic, which might eventually benefit from the increasing availability of high throughput technologies, including metagenomic analysis.

## Author Contributions

MC, RG, and PM conceived the idea and outline of the review. All authors contributed to writing specific sections and approved the final version of the manuscript.

## Conflict of Interest Statement

The authors declare that the research was conducted in the absence of any commercial or financial relationships that could be construed as a potential conflict of interest.

## References

[B1] Aceituno F. F. Orellana M. Torres J. Mendoza S. Slater A. W. Melo F. (2012). Oxygen response of the wine yeast *Saccharomyces cerevisiae* EC1118 grown under carbon-sufficient, nitrogen-limited enological conditions. *Appl. Environ. Microbiol.* 78 8340–8352. 10.1128/AEM.02305-12 23001663 PMC3497381

[B2] Alexander M. A. Jeffries T. W. (1990). Respiratory efficiency and metabolite partitioning as regulatory phenomena in yeast. *Enzyme Microb. Technol.* 12 2–19. 10.1016/0141-0229(90)90173-N

[B3] Alexandre H. Charpentier C. (1998). Biochemical aspects of stuck and sluggish fermentation in grape must. *J. Ind. Microbiol. Biotechnol.* 20 20–27. 10.1038/sj.jim.2900442

[B4] Alonso A. Belda I. Santos A. Navascues E. Marquina D. (2015). Advances in the control of the spoilage caused by *Zygosaccharomyces* species on sweet wines and concentrated grape musts. *Food Control* 51 129–134. 10.1016/j.foodcont.2014.11.019

[B5] Alston J. M. Fuller K. B. James T. Lapsley J. T. Soleas G. (2011). Too much of a good thing? Causes and consequences of increases in sugar content of California wine grapes. *J. Wine Econom.* 6 135–159. 10.1017/S1931436100001565

[B6] Alston J. M. Fuller K. B. Lapsley J. T. Soleas G. Tumber K. P. (2015). Splendide mendax: false label claims about high and rising alcohol content of wine. *J. Wine Econom.* 10 275–313. 10.1017/jwe.2015.33

[B7] Anfang N. Brajkovich M. Goddard M. R. (2009). Co-fermentation with *Pichia kluyveri* increases varietal thiol concentrations in Sauvignon Blanc. *Aust. J. Grape Wine Res.* 15 1–8. 10.1111/j.1755-0238.2008.00031.x

[B8] Arneborg N. Siegumfeldt H. Andersen G. H. Nissen P. Daria V. R. Rodrigo P. G. (2005). Interactive optical trapping shows that confinement is a determinant of growth in a mixed yeast culture. *FEMS Microbiol. Lett* 245 155–159. 10.1016/j.femsle.2005.03.008 15796993

[B9] Barbosa C. Mendes-Faia A. Lage P. Mira N. P. Mendes-Ferreira A. (2015). Genomic expression program of *Saccharomyces cerevisiae* along a mixed-culture wine fermentation with *Hanseniaspora guilliermondii*. *Microb. Cell Fact.* 14:124 10.1186/s12934-015-0318-1 PMC4552253 26314747

[B10] Barnett J. A. Entian K. D. (2005). A history of research on yeasts 9: regulation of sugar metabolism. *Yeast* 22 835–894. 10.1002/yea.1249 16134093

[B11] Barwald G. Fischer A. (1996). Crabtree effect in aerobic fermentations using grape juice for the production of alcohol reduced wine. *Biotechnol. Lett.* 18 1187–1192. 10.1007/BF00128590

[B12] Belda I. Navascués E. Marquina D. Santos A. Calderon F. Benito S. (2015). Dynamic analysis of physiological properties of *Torulaspora delbrueckii* in wine fermentations and its incidence on wine quality. *Appl. Microbiol. Biotechnol.* 99 1911–1922. 10.1007/s00253-014-6197-2 25408314

[B13] Belda I. Ruiz J. Alastruey-Izquierdo A. Navascués E. Marquina D. Santos A. (2016). Unraveling the enzymatic basis of wine “flavorome”: a phylo-functional study of wine related yeast species. *Front. Microbiol.* 7:12 10.3389/fmicb.2016.00012 PMC4718978 26834730

[B14] Bely M. Stoeckle P. Masneuf-Pomarède I. Dubourdieu D. (2008). Impact of mixed torulaspora delbrueckii–*Saccharomyces cerevisiae* culture on high-sugar fermentation. *Int. J. Food Microbiol.* 122 312–320. 10.1016/j.ijfoodmicro.2007.12.023 18262301

[B15] Bindon K. Varela C. Kennedy J. Holt H. Herderich M. (2013). Relationships between harvest time and wine composition in *Vitis vinifera* L. cv. Cabernet Sauvignon. grape and wine chemistry. *Food Chem.* 138 1696–1705. 10.1016/j.foodchem.2012.09.146 23411300

[B16] Branco P. Francisco D. Chambon C. Hébraud M. Arneborg N. Almeida M. G. (2014). Identification of novel GAPDH-derived antimicrobial peptides secreted by *Saccharomyces cerevisiae* and involved in wine microbial interactions. *Appl. Microbiol. Biotechnol.* 98 843–853. 10.1007/s00253-013-5411-y 24292082

[B17] Cadière A. Ortiz-Julien A. Camarasa C. Dequin S. (2011). Evolutionary engineered *Saccharomyces cerevisiae* wine yeast strains with increased in vivo flux through the pentose phosphate pathway. *Metab. Eng.* 13 263–271. 10.1016/j.ymben.2011.01.008 21300171

[B18] Cambon B. Monteil V. Remize F. Camarasa C. Dequin S. (2006). Effects of GPD1 overexpression in *Saccharomyces cerevisiae* commercial wine yeast strains lacking ALD6 genes. *Appl. Environ. Microbiol.* 72 4688–4694. 10.1128/AEM.02975-05 16820460 PMC1489326

[B19] Canonico L. Comitini F. Oro L. Ciani M. (2016). Sequential fermentation with selected immobilized non-*Saccharomyces* yeast for reduction of ethanol content in wine. *Front. Microbiol.* 7:278 10.3389/fmicb.2016.00278 PMC4786567 27014203

[B20] Cheraiti N. Guezenec S. Salmon J. M. (2005). Redox interactions between *Saccharomyces cerevisiae* and *Saccharomyces uvarum* in mixed culture under enological conditions. *Appl. Environ. Microbiol.* 71 255–260. 10.1128/AEM.71.1.255-260.2005 15640195 PMC544210

[B21] Ciani M. Beco L. Comitini F. (2006). Fermentation behaviour and metabolic interactions of multistarter wine yeast fermentations. *Int. J. Food Microbiol.* 108 239–245. 10.1016/j.ijfoodmicro.2005.11.012 16487611

[B22] Ciani M. Comitini F. (2015). Yeast interactions in multi-starter wine fermentation. *Curr. Opin. Food Sci.* 1 1–6. 10.1016/j.cofs.2014.07.001

[B23] Ciani M. Comitini F. Mannazzu I. Domizio P. (2010). Controlled mixed culture fermentation: a new perspective on the use of non-*Saccharomyces* yeasts in winemaking. *FEMS Yeast Res.* 10 123–133. 10.1111/j.1567-1364.2009.00579.x 19807789

[B24] Ciani M. Ferraro F. Fatichenti F. (2000). Influence of glycerol production on the aerobic and anaerobic growth of the wine yeast *Candida stellata*. *Enzyme Microb. Technol.* 27 698–703. 10.1016/S0141-0229(00)00269-6 11064052

[B25] Ciani M. Ferraro L. (1996). Enhanced glycerol content in wines made with immobilized *Candida stellata* cells. *Appl. Environ. Microb.* 62 128–132. 10.1128/aem.62.1.128-132.1996 PMC1388745 16535203

[B26] Ciani M. Ferraro L. (1998). Combined use of immobilised *Candida stellata* cells and *Saccharomyces cerevisiae* to improve the quality of wines. *J. Appl. Bacteriol.* 85 247–254. 10.1046/j.1365-2672.1998.00485.x 9750297

[B27] Ciani M. Maccarelli F. (1998). Oenological properties of non-*Saccharomyces* yeasts associated with wine-making. *World J. Microbiol. Biotechnol.* 14 199–203. 10.1023/A:1008825928354

[B28] Ciani M. Picciotti G. (1995). The growth kinetics and fermentation behaviour of some non-*Saccharomyces* yeasts associated with wine-making. *Biotechnol. Lett.* 17 1247–1250. 10.1007/BF00128395

[B29] Ciani M. Rosini G. (1995). Validity of Genevois’ equation in wine yeast selection. *Ann. Microbiol. Enzim.* 45 201–207.

[B30] Comitini F. Gobbi M. Domizio P. Romani C. Lencioni L. Mannazzu I. (2011). Selected non-*Saccharomyces* wine yeasts in controlled multistarter fermentations with *Saccharomyces cerevisiae*. *Food Microbiol.* 28 873–882. 10.1016/j.fm.2010.12.001 21569929

[B31] Contreras A. Curtin C. Varela C. (2015a). Yeast population dynamics reveal a potential ‘collaboration’ between *Metschnikowia pulcherrima* and *Saccharomyces* uvarum for the production of reduced alcohol wines during Shiraz fermentation. *Appl. Microbiol. Biotechnol.* 99 1885–1895. 10.1007/s00253-014-6193-6 25388943

[B32] Contreras A. Hidalgo C. Schmidt S. Henschke P. A. Curtin C. Varela C. (2015b). The application of non-*Saccharomyces* yeast in fermentations with limited aeration as a strategy for the production of wine with reduced alcohol content. *Int. J. Food Microbiol.* 205 7–15. 10.1016/j.ijfoodmicro.2015.03.027 25866906

[B33] Contreras A. Hidalgo C. Schmidt S. Henschke P. A. Curtin C. Varela C. (2014). Evaluation of non-*Saccharomyces* yeasts for the reduction of alcohol content in wine. *Appl. Environ. Microbiol.* 80 1670–1678. 10.1128/AEM.03780-13 24375129 PMC3957604

[B34] Coulter A. D. Henschke P. A. Simos C. A. Pretorius I. S. (2008). When the heat is on, yeast fermentation runs out of puff. *Aust. N. Z. Wine Ind. J.* 23 26–30.

[B35] de Deken R. H. (1966). The crabtree effect: a regulatory system in yeast. *J. Gen. Microbiol.* 44 149–156. 10.1099/00221287-44-2-149 5969497

[B36] Di Maio S. Genna G. Gandolfo V. Amore G. Ciaccio M. Oliva D. (2012). Presence of *Candida zemplinina* in Sicilian musts and selection of a strain for wine mixed fermentations. *S. Afr. J. Enol. Vitic.* 33 80–87.

[B37] Domizio P. Lencioni L. Ciani M. Di Blasi S. Pontremolesi C. Sabatelli M. (2007). Spontaneous and inoculated yeast population dynamics and their effect on organoleptic characters of Vinsanto wine under different process conditions. *Int. J. Food Microbiol.* 115 281–289. 10.1016/j.ijfoodmicro.2006.10.052 17307268

[B38] Domizio P. Romani C. Lencioni L. Comitini F. Gobbi M. Mannazzu I. (2011). Outlining a future for non-*Saccharomyces* yeasts: selection of putative spoilage wine strains to be used in association with *Saccharomyces cerevisiae* for grape juice fermentation. *Int. J. Food Microbiol.* 147 170–180. 10.1016/j.ijfoodmicro.2011.03.020 21531033

[B39] Eglinton J. M. Heinrich A. J. Pollnitz A. P. Langridge P. Henschke P. A. Lopes M. D. (2002). Decreasing acetic acid accumulation by a glycerol overproducing strain of *Saccharomyces cerevisiae* by deleting the ALD6 aldehyde dehydrogenase gene. *Yeast* 19 295–301. 10.1002/yea.834 11870853

[B40] Ehsani M. Fernandez M. R. Biosca J. A. Julien A. Dequin S. (2009). Engineering of 2,3-butanediol dehydrogenase to reduce acetoin formation by glycerol-overproducing, low-alcohol *Saccharomyces cerevisiae*. *Appl. Environ. Microbiol.* 75 3196–3205. 10.1128/aem.02157-08 19329666 PMC2681661

[B41] Erten H. Campbell I. (2001). The production of low-alcohol wines by aerobic yeasts. *J. Inst. Brew.* 107 207–215. 10.1002/j.2050-0416.1953.tb06929

[B42] Escudero A. Campo E. Fariña L. Cacho J. Ferreira V. (2007). Analytical characterization of the aroma of five premium red wines. Insights into the Role of Odor Families and the Concept of Fruitiness of Wines. *J. Agric. Food Chem.* 55 4501–4510. 10.1021/jf0636418 17488088

[B43] Ferraro L. Fatichenti F. Ciani M. (2000). Pilot scale vinification process by immobilised *Candida stellata* and *Saccharomyces cerevisiae*. *Process. Biochem.* 35 1125–1129. 10.1016/S0032-9592(00)00148-5

[B44] Fischer U. (2010). Sensorische bedeutung des alkohols im wein. Eine unterschätzte Einflussgröße. *Das Deutsche Weinmagazin* 19 108–110.

[B45] Fleet G. H. (2008). Wine yeasts for the future. *FEMS Yeast Res.* 8 979–995. 10.1111/j.1567-1364.2008.00427.x 18793201

[B46] Fornairon-Bonnefond C. Aguera E. Deytieux C. Sablayrolles J. M. Salmon J. M. (2003). Impact of oxygen addition during enological fermentation on sterol contents in yeast lees and their reactivity towards oxygen. *J. Biosci. Bioeng.* 95 496–503. 10.1016/S1389-1723(03)80051-8 16233446

[B47] Fornairon-Bonnefond C. Demaretz V. Rosenfeld E. Salmon J. M. (2002). Oxygen addition and sterol synthesis in *Saccharomyces cerevisiae* during enological fermentation. *J. Biosci. Bioeng.* 93 176–182. 10.1016/S1389-1723(02)80011-1 16233184

[B48] Frost R. Quiñones I. Veldhuizen M. Alava J. I. Small D. Carreiras M. (2015). What can the brain teach us about winemaking? An fMRI study of alcohol level preferences. *PLoS ONE* 10:e0119220 10.1371/journal.pone.0119220 PMC4364721 25785844

[B49] Gambuti A. Rinaldi A. Lisanti M. T. Pessina R. Moio L. (2011). Partial dealcoholisation of red wines by membrane contactor technique: influence on colour, phenolic compounds and saliva precipitation index. *Eur. Food Res. Technol.* 233 647–655. 10.1007/s00217-011-1553-2

[B50] Gancedo C. Serrano R. (1989). “Energy yielding metabolism,” in *The Yeasts*, 2nd Edn Vol. 3 eds Rose A. H. Harrison J. S. (London: Academic Press), 205–259.

[B51] García-Martín N. Pérez-Magariño S. Ortega-Heras M. González-Huerta C. Mihnea M. González-San José M. L. (2010). Sugar reduction in musts with nanofiltration membranes to obtain low alcohol-content wines. *Sep. Purif. Technol.* 76 158–170. 10.1016/j.seppur.2010.10.002

[B52] Gil M. Estevez S. Kontoudakis N. Fort F. Canals J. M. Zamora F. (2013). Influence of partial dealcoholization by reverse osmosis on red wine composition and sensory characteristics. *Eur. Food Res. Technol.* 237 481–488. 10.1007/s00217-013-2018-6

[B53] Giovanelli G. Peri C. Parravicini E. (1996). Kinetics of grape juice fermentation under aerobic and anaerobic conditions. *Am. J. Enol. Vitic.* 47 429–434.

[B54] Giudici P. Zambonelli C. Passarelli P. Castellari L. (1995). Improvement of wine composition with cryotolerant *Saccharomyces* strains. *Am. J. Enol. Vitic.* 46 143–147.

[B55] Gobbi M. Comitini F. Domizio P. Romani C. Lencioni L. Mannazzu I. (2013). *Lachancea thermotolerans* and *Saccharomyces cerevisiae* in simultaneous and sequential co-fermentation: a strategy to enhance acidity and improve the overall quality of wine. *Food Microbiol.* 33 271–281. 10.1016/j.fm.2012.10.004 23200661

[B56] Gobbi M. De Vero L. Solieri L. Comitini F. Oro L. Giudici P. (2014). Fermentative aptitude of non-*Saccharomyces* wine yeast for reduction in the ethanol content in wine. *Eur. Food. Res. Technol.* 239 41–48. 10.1007/s00217-014-2187-y

[B57] Gonzalez R. Quirós M. Morales P. (2013). Yeast respiration of sugars by non-*Saccharomyces* yeast species: a promising and barely explored approach to lowering alcohol content of wines. *Trends Food Sci. Technol.* 29 55–61. 10.1016/j.tifs.2012.06.015

[B58] Grant M. (2010). “Who is listening?,” in *Proceedings of Fourteenth Australian Wine Industry Technical Conference*, Adelaide, SA: AWITC Inc, 25–27.

[B59] Harbertson J. F. Mireles M. S. Harwood E. D. Weller K. M. Ross C. F. (2009). Chemical and sensory effects of saignee, water addition, and extended maceration on high Brix must. *Am. J. Enol. Vitic.* 60 450–460.

[B60] Heard G. M. (1999). Novel yeasts in winemaking—looking to the future. *Food Aust.* 51 347–352.

[B61] Holzer H. (1961). Regulation of carbohydrate metabolism by enzyme competition. *Cold. Spring Harb. Symp. Quant. Biol.* 26 277–288. 10.1101/SQB.1961.026.01.034 13908636

[B62] Hu K. Zhu X. L. Mu H. Ma Y. Ullah N. Tao Y. S. (2016). A novel extracellular glycosidase activity from *Rhodotorula mucilaginosa*: its application potential in wine aroma enhancement. *Lett. Appl. Microbiol.* 62 169–176. 10.1111/lam.12527 26606736

[B63] Jansen M. L. A. Diderich J. A. Mashego M. Hassane A. de Winde J. H. Daran-Lapujade P. (2005). Prolonged selection in aerobic, glucose-limited chemostat cultures of *Saccharomyces cerevisiae* causes a partial loss of glycolytic capacity. *Microbiology* 151 1657–1669. 10.1099/mic.0.27577-0 15870473

[B64] Jolly N. P. Augustyn O. P. H. Pretorius I. S. (2006). The role and use of non-*Saccharomyces* yeasts in wine production. *S. Afr. J. Enol. Vitic.* 27 15–38.

[B65] Jolly N. P. Varela C. Pretorius I. S. (2014). Not your ordinary yeast: non-*Saccharomyces* yeasts in wine production uncovered. *FEMS Yeast Res.* 14 215–237. 10.1111/1567-1364.12111 24164726

[B66] Jones G. V. White M. A. Cooper O. R. Storchmann K. (2005). Climate change and global wine quality. *Clim. Change* 73 319–343. 10.1007/s10584-005-4704-2

[B67] Kemsawasd V. Viana T. Ardö Y. Arneborg N. (2015). Influence of nitrogen sources on growth and fermentation performance of different wine yeast species during alcoholic fermentation. *Appl. Microbiol. Biotechnol.* 99 10191–10207. 10.1007/00253-015-6835-3 26257263

[B68] Kishimoto M. Oshida A. Shinohara T. Soma E. Goto S. (1994). Effect of temperature on ethanol productivity and resistance of cryophilic wine yeasts. *J. Gen. Appl. Microbiol.* 40 135–142. 10.2323/jgam.40.135

[B69] Konig H. Unden G. Frolich J. (2009). *Biology of Microorganisms on Grapes, in Must and in Wine*. Heidelberg: Springer.

[B70] Kontoudakis N. Esteruelas M. Fort F. Canals J. M. Zamora F. (2011). Use of unripe grapes harvested during cluster thinning as a method for reducing alcohol content and pH of wine. *Aust. J. Grape Wine Res.* 17 230–238. 10.1111/j.1755-0238.2011.00142.x

[B71] Kurtzman C. P. Fell J. W. Boekhout T. (2011). *The Yeasts, a Taxonomic Study*. Amsterdam: Elsevier.

[B72] Kutyna D. R. Varela C. Henschke P. A. Chambers P. A. Stanley G. A. (2010). Microbiological approaches to lowering ethanol concentration in wine. *Trends Food Sci. Technol.* 21 293–302. 10.1016/j.tifs.2010.03.004

[B73] Kutyna D. R. Varela C. Henschke P. A. Chambers P. J. Stanley G. A. (2012). Adaptive evolution of *Saccharomyces cerevisiae* to generate strains with enhanced glycerol production. *Appl. Microbiol. Biotechnol.* 93 1175–1184. 10.1007/s00253-011-3622-7 21989563

[B74] Lage P. Barbosa C. Mateus B. Vasconcelos I. Mendes-Faia A. Mendes-Ferreira A. (2014). H. guilliermondii impacts growth kinetics and metabolic activity of *S. cerevisiae*: the role of initial nitrogen concentration. *Int. J. Food Microbiol.* 172 62–69. 10.1016/j.ijfoodmicro.2013.11.031 24361834

[B75] Lambrechts M. G. Pretorius I. S. (2000). Yeast and its importance to wine aroma. *S. Afr. J. Enol. Vitic.* 21 97–129.

[B76] Lopes C. A. Rodríguez M. E. Querol A. Bramardi S. Caballero A. C. (2006). Relationship between molecular and enological features of Patagonian wine yeasts:relevance in selection protocols. *World J. Microbiol. Biotechnol.* 22 827–833. 10.1007/s11274-005-9110-4

[B77] MacAvoy M. G. (2010). “Wine—harmful or healthy? What is being considered in Australia and New Zealand?,” in *Proceedings of Fourteenth Australian Wine Industry Technical Conference*. Glen Osmond, SA: AWITC Inc.

[B78] Magyar I. Toth T. (2011). Comparative evaluation of some oenological properties in wine strains of *Candida stellata*, *Candida zemplinina*, *Saccharomyces uvarum* and *Saccharomyces cerevisiae*. *Food Microbiol.* 28 94–100. 10.1016/j.fm.2010.08.011 21056780

[B79] Martinez de Toda F. Sancha J. C. Balda P. (2013). Reducing the sugar and pH of the grape (*Vitis vinifera* L. cvs. ‘Grenache’ and ‘Tempranillo’) through a single shoot trimming. *S. Afr. J. Enol. Vitic.* 34 246–251.

[B80] Masneuf-Pomarede I. Bely M. Marullo P. Albertin W. (2016). The genetics of non-conventional wine yeasts: current knowledge and future challenges. *Front. Microbiol.* 6:1563 10.3389/fmicb.2015.01563 PMC4707289 26793188

[B81] Masneuf-Pomarede I. Bely M. Marullo P. Lonvaud-Funel A. Dubourdieu D. (2010). Reassessment of phenotypic traits for *Saccharomyces bayanus* var. uvarum wine yeast strains. *Int. J. Food Microbiol.* 139 79–86. 10.1016/j.ijfoodmicro.2010.01.038 20188428

[B82] Medina K. Boido E. Dellacassa E. Carrau F. (2012). Growth of non-*Saccharomyces* yeasts affects nutrient availability for *Saccharomyces cerevisiae* during wine fermentation. *Int. J. Food Microbiol.* 157 245–250. 10.1016/j.ijfoodmicro.2012.05.012 22687186

[B83] Medina K. Boido E. Fariña L. Dellacassa E. Carrau F. (2016). Non-*Saccharomyces* and *Saccharomyces* strains co-fermentation increases acetaldehyde accumulation. Effect on anthocyanin derived pigments in Tannat red wines. *Yeast* 10.1002/yea.3156 [Epub ahead of print]. 26888345

[B84] Meillon S. Urbano C. Guillot G. Schlich P. (2010a). Acceptability of partially dealcoholized wines—measuring the impact of sensory and information cues on overall liking in real-life settings. *Food Qual. Prefer.* 21 763–773. 10.1016/j.foodqual.2010.07.013

[B85] Meillon S. Viala D. Medel M. Urbano C. Guillot G. Schlich P. (2010b). Impact of partial alcohol reduction in Syrah wine on perceived complexity and temporality of sensations and link with preference. *Food Qual. Prefer.* 21 732–740. 10.1016/j.foodqual.2010.06.005

[B86] Mendez-Costabel M. (2007). *Impact of Irrigation Levels During the Latter Stages of Fruit Ripening on the Yield Components, Physiology and Berry and Wine Composition of Vitis vinifera L*. M.S thesis, University of California, Davis, CA.

[B87] Michnick S. Roustan J. L. Remize F. Barre P. Dequin S. (1997). Modulation of glycerol and ethanol yields during alcoholic fermentation in *Saccharomyces cerevisiae* strains overexpressed or disrupted for GPD1 encoding glycerol 3-phosphate dehydrogenase. *Yeast* 13 783–793. 10.1002/(SICI)1097-0061(199707)13:9<783::AID-YEA128>3.0.CO;2-W 9234667

[B88] Milanovic V. Ciani M. Oro L. Comitini F. (2012). *Starmerella bombicola* influences the metabolism of *Saccharomyces cerevisiae* at pyruvate decarboxylase and alcohol dehydrogenase level during mixed wine fermentation. *Microb. Cell Fact.* 11 18 10.1186/1475-2859-11-18 PMC3295710 22305374

[B89] Mira de Orduña R. (2010). Climate change associated effects on grape and wine quality and production. *Food Res. Int.* 43 1844–1855. 10.1016/j.foodres.2010.05.001

[B90] Morales P. Rojas V. Quirós M. Gonzalez R. (2015). The impact of oxygen on the final alcohol content of wine fermented by a mixed starter culture. *Appl. Microbiol. Biotechnol.* 99 3993–4003. 10.1007/s00253-014-6321-3 25582558 PMC4428804

[B91] Muller-Thurgau L. (1896). Uber den ursprung der weinhefe und hieran sich knuepfende praktische folgerungen. *Weinbau Weinhandel* 7 40–41.

[B92] Oro L. Ciani M. Comitini F. (2014). Antimicrobial activity of *Metschnikowia pulcherrima* on wine yeasts. *J. Appl. Microbiol.* 116 1209–1217. 10.1111/jam.12446 24443784

[B93] Otterstedt K. Larsson C. Bill R. M. Stahlberg A. Boles E. Hohmann S. (2004). Switching the mode of metabolism in the yeast *Saccharomyces cerevisiae*. *EMBO Rep* 5 532–537. 10.1038/sj.embor.7400132 15071495 PMC1299050

[B94] Papini M. Nookaew I. Uhlén M. Nielsen J. (2012). Scheffersomyces stipitis: a comparative systems biology study with the Crabtree positive yeast *Saccharomyces cerevisiae*. *Microb. Cell Fact.* 11 136 10.1186/1475-2859-11-136 PMC3528450 23043429

[B95] Pickering G. J. (2000). Low- and reduced-alcohol wine: a review. *J. Wine Res.* 11 129–144. 10.1080/09571260020001575

[B96] Piskur J. Rozpedowska E. Polakova S. Merico A. Compagno C. (2006). How did *Saccharomyces* evolve to become a good brewer? *Trends Genet.* 22 183–186. 10.1016/j.tig.2006.02.002 16499989

[B97] Poni S. (2014). The impact of leaf removal and the management of crop load on fruit quality - a European perspective. *Wine Vitic. J.* 29 44–48.

[B98] Pretorius I. S. Hoj P. B. (2005). Grape and wine biotechnology: challenges, opportunities and potential benefits. *Aust. J. Grape Wine Res.* 11 83–108. 10.1111/j.1755-0238.2005.tb00281.x

[B99] Pronk J. T. Steensma H. Y. van Dijken J. P. (1996). Pyruvate metabolism in *Saccharomyces cerevisiae*. *Yeast* 12 1607–1633. 10.1002/(SICI)1097-0061(199612)12:16<1607::AID-YEA70>3.0.CO;2-4 9123965

[B100] Quirós M. Rojas V. Gonzalez R. Morales P. (2014). Selection of non-*Saccharomyces* yeast strains for reducing alcohol levels in wine by sugar respiration. *Int. J. Food Microbiol.* 181 85–91. 10.1016/j.ijfoodmicro.2014.04.024 24831930

[B101] Remize F. Andrieu E. Dequin S. (2000). Engineering of the pyruvate dehydrogenase bypass in *Saccharomyces cerevisiae*: role of the cytosolic Mg2+ and mitochondrial K+ acetaldehyde dehydrogenases Ald6p and Ald4p in acetate formation during alcoholic fermentation. *Appl. Environ. Microbiol.* 66 3151–3159. 10.1128/AEM.66.8.3151-3159.2000 10919763 PMC92127

[B102] Remize F. Roustan J. L. Sablayrolles J. M. Barre P. Dequin S. (1999). Glycerol overproduction by engineered *Saccharomyces cerevisiae* wine yeast strains leads to substantial changes in by-product formation and to a stimulation of fermentation rate in stationary phase. *Appl. Environ. Microbiol.* 65 143–149. 9872772 10.1128/aem.65.1.143-149.1999 PMC90995

[B103] Renouf V. Claisse O. Lonvaud-Funel A. (2007). Inventory and monitoring of wine microbial consortia. *Appl. Microbiol. Cell Physiol.* 75 149–164. 10.1007/s00253-006-0798-3 17235561

[B104] Ribéreau-Gayon J. Peynaud E. (1960). *Traité d’śnologie*. Paris: Paris et Liége Librarie Polytechnique Ch. Bèranger. 293–298.

[B105] Robinson A. L. Ebeler S. E. Heymann H. Boss P. K. Solomon P. S. Trengove R. D. (2009). Interactions between wine volatile compounds and grape and wine matrix components influence aroma compound headspace partitioning. *J. Agric. Food Chem.* 57 10313–10322. 10.1021/jf902586n 19845354

[B106] Rodrigues A. J. Raimbaud T. Gonzalez R. Morales P. (2016). Environmental factors influencing the efficacy of different yeast strains for alcohol level reduction in wine by respiration. *LWT Food Sci. Technol.* 65 1038–1043. 10.1016/j.lwt.2015.09.046

[B107] Rojas V. Gil J. V. Pinaga F. Manzanares P. (2001). Studies on acetate ester production by non-*Saccharomyces* wine yeasts. *Int. J. Food Microbiol.* 70 283–289. 10.1016/S0168-1605(01)00552-9 11764193

[B108] Rojas V. Gil J. V. Piñaga F. Manzanares P. (2003). Acetate ester formation in wine by mixed cultures in laboratories fermentations. *Int. J. Food Microbiol.* 86 181–188. 10.1016/S0168-1605(03)00255-1 12892933

[B109] Romano P. Fiore C. Paraggio M. Caruso M. Capece A. (2003). Function of yeasts species and strains in wine flavour. *Int. J. Food Microbiol.* 86 169–180. 10.1016/S0168-1605(03)00290-3 12892932

[B110] Romano P. Suzzi G. Zironi R. Comi G. (1992). Biometric study of acetoin production in *Hanseniaspora guilliermondii* and *Kloeckera apiculata*. *Appl. Environ. Microbiol.* 59 1838–1841. 16348961 10.1128/aem.59.6.1838-1841.1993 PMC182169

[B111] Sadoudi M. Tourdot-Marechal R. Rousseaux S. Steyer D. Gallardo- Chacon J. J. Ballester J. (2012). Yeast-yeast interactions revealed by aromatic profile analysis of Sauvignon Blanc wine fermented by single or co-culture of non-*Saccharomyces* and *Saccharomyces* yeasts. *Food Microbiol.* 32 243–253. 10.1016/j.fm.2012.06.006 22986187

[B112] Saliba A. Ovington L. Moran C. C. Bruwer J. (2013). Consumer attitudes to low alcohol wine: an australian sample. *Wine Vitic. J.* 28 58–61.

[B113] Salmon J. (2006). Interactions between yeast, oxygen and polyphenols during alcoholic fermentations: practical implications. *LWT Food Sci. Technol.* 39 959–965. 10.1016/j.lwt.2005.11.005

[B114] Schmidtke L. M. Blackman J. W. Agboola S. O. (2012). Production technologies for reduced alcoholic wines. *J. Food Sci.* 77 25–41. 10.1111/j.1750-3841.2011.02448.x 22260123

[B115] Sipiczki M. Ciani M. Csoma H. (2005). Taxonomic reclassification of *Candida stellata* DBVPG 3827. *Folia Microbiol.* 50 494–498. 10.1007/BF02931436 16681146

[B116] Smith P. M. (1995). *Biological Processes for the Reduction of Alcohol in Wines.* MASc., dissertation, Lincoln University, Lincoln.

[B117] Swiegers J. H. Bartowsky E. J. Henschke P. A. Pretorius I. S. (2005). Yeast and bacterial modulation of wine aroma and flavor. *Aust. J. Grape Wine Res.* 11 139–173. 10.1111/j.1755-0238.2005.tb00285.x

[B118] Symons G. M. Davies C. Shavrukov Y. Dry I. B. Reid J. B. Thomas M. R. (2006). Grapes on steroids. Brassinosteroids are involved in grape berry ripening. *Plant Physiol.* 140 150–158. 10.1104/pp.104.900182 16361521 PMC1326039

[B119] Teissedre P. L. (2013). “Alcohol level reduction in wine,” in *Proceedings of the 1st International Symposium Oenoviti International Network*, Villenave d’Ornon: Vigne et Vin Publications Internationales.

[B120] Tofalo R. Schirone M. Torriani S. Rantsiou K. Cocolin L. Perpetuini G. (2012). Diversity of *Candida zemplinina* strains from grapes and Italian wines. *Food Microbiol.* 29 18–26. 10.1016/j.fm.2011.08.014 22029914

[B121] Valero E. Millan C. Ortega J. M. (2001). Influence of oxygen addition during growth phase on the biosynthesis of lipids in *Saccharomyces cerevisiae* (M330-9) in enological fermentations. *J. Biosci. Bioeng.* 92 33–38. 10.1016/S1389-1723(01)80195-X 16233054

[B122] Varela C. Dry P. R. Kutyna D. R. Francis I. L. Henschke P. A. Curtin C. D. (2015). Strategies for reducing alcohol concentration in wine. *Aust. J. Grape Wine Res* 21 670–679. 10.1111/ajgw.12187

[B123] Varela C. Kutyna D. R. Solomon M. R. Black C. A. Borneman A. Henschke P. A. (2012). Evaluation of gene modification strategies for the development of low-alcohol-wine yeasts. *Appl. Environ. Microbiol.* 78 6068–6077. 10.1128/AEM.01279-12 22729542 PMC3416606

[B124] Viana F. Gil J. V. Genovés S. Vallés S. Manzanares P. (2008). Rational selection of non-*Saccharomyces* wine yeasts for mixed starters based on ester formation and enological traits. *Food Microbiol.* 25 778–785. 10.1016/j.fm.2008.04.015 18620969

[B125] Wang C. Mas A. Esteve-Zarzoso B. (2015). Interaction between *Hanseniaspora uvarum* and *Saccharomyces cerevisiae* during alcoholic fermentation. *Int. J. Food Microbiol.* 206 67–74. 10.1016/j.ijfoodmicro.2015.04.022 25956738

